# Cardiometabolic Risk Factors, Obesity Phenotypes, and Novel Anthropometric Indices in Patients With Acute Coronary Syndrome: A Hospital-Based Case-Control Study in Central India

**DOI:** 10.7759/cureus.116416

**Published:** 2026-09-17

**Authors:** Sanskruti Boob, Tanuja Manohar, Fahad Idrees Shaikh, Yash Dad, Ibtehsam Ather Hussain, Rehmat F Varawalla, Masood Ali Mohammed, Shaestha Khatoon, Malvika Chandwani

**Affiliations:** 1 General Medicine, Lata Mangeshkar Hospital, N.K.P. Salve Institute of Medical Sciences and Research Centre, Nagpur, IND; 2 Medicine, Lata Mangeshkar Hospital, N.K.P. Salve Institute of Medical Sciences and Research Centre, Nagpur, IND; 3 Dermatology Venereology and Leprosy, Kalinga Institute of Medical Sciences, Bhubaneshwar, IND; 4 Medicine, Deccan College of Medical Sciences, Hyderabad, IND; 5 General Medicine, Deccan College of Medical Sciences, Hyderabad, IND; 6 Medicine, TRR Institute of Medical Sciences, Hyderabad, IND; 7 Medicine, Sri Aurobindo Institute of Medical Sciences, Ujjain, IND

**Keywords:** acute coronary syndrome, anthropometric indices, body roundness index, cardiometabolic risk factors, case-control study, obesity phenotype, waist-to-height ratio

## Abstract

Background: Acute coronary syndrome (ACS) is the most severe manifestation of cardiovascular disease and disproportionately affects South Asians at a younger age than Western populations. Body mass index (BMI) inadequately reflects visceral adiposity, particularly in Indian populations. Obesity phenotypes, which combine BMI with metabolic status, and novel anthropometric indices may improve cardiovascular risk assessment, but data from Indian ACS patients remain limited.

Objectives: To evaluate the association between cardiometabolic risk factors, obesity phenotypes, and novel anthropometric indices with ACS, and to quantify these associations using odds ratios (ORs).

Methods: This case-control study enrolled 50 patients with confirmed ACS (unstable angina, Non-ST-elevation myocardial infarction (NSTEMI), or ST-elevation myocardial infarction (STEMI)) and 50 age-, sex-, and weight-matched healthy controls at a tertiary care hospital. Sociodemographic, clinical, anthropometric, and biochemical data were recorded. Participants were classified into four obesity phenotypes - metabolically healthy normal weight (MHNW), metabolically unhealthy normal weight (MUNW), metabolically healthy overweight/obese (MHO), and metabolically unhealthy overweight/obese (MUO) - based on BMI and presence of hypertension, diabetes, and/or dyslipidemia. Five novel indices were calculated: A body shape index (ABSI), body roundness index (BRI), waist-to-height ratio (WHtR), weight-adjusted waist index (WWI), and abdominal volume index (AVI). Continuous variables were compared using t-tests and categorical variables using chi-square/Fisher's exact test; unadjusted ORs with 95% CIs were calculated. p<0.05 was significant. ST-elevation myocardial infarction

Results: Cases and controls were comparable for age and sex (p>0.05). Hypertension (60% vs 36%), diabetes (52% vs 28%), dyslipidemia (56% vs 32%), and smoking (44% vs 24%) were more common among cases (all p<0.05). Per 1-SD increase, BMI was not significantly associated with ACS (Exp(B) 1.208, 95% CI 0.816-1.790, Wald 0.89, p=0.345), whereas waist circumference, waist-to-hip ratio, and neck circumference were each significantly associated with ACS. Each of the five novel indices was significantly associated with ACS per 1-SD increase (all p<0.001; e.g., WHtR Exp(B) 2.473, 95% CI 1.638-3.732, Wald 18.59); because each index reflects a different geometric construct even when expressed per 1-SD, indices are not ranked by strength of association. Using MHNW as reference, MUO showed significantly increased odds of ACS (OR 3.33, 95% CI 1.10-10.12); MUNW showed a similar point estimate (OR 3.33, 95% CI 0.89-12.49) that did not reach significance (p=0.074), and MHO was not significantly associated with ACS (OR 1.50, 95% CI 0.48-4.65). All ORs are unadjusted.

Conclusion: In this unadjusted, cross-sectional comparison, metabolic health and central fat distribution were associated with ACS, whereas BMI was not. Obesity phenotyping and novel anthropometric indices warrant further evaluation in larger, multivariable-adjusted, prospective studies as potential adjuncts to cardiovascular risk stratification in South Asian populations.

## Introduction

Cardiovascular diseases (CVDs) are the leading cause of death worldwide, accounting for nearly one-third of all deaths globally [1]. Acute coronary syndrome (ACS) - encompassing unstable angina, non-ST-elevation myocardial infarction (NSTEMI), and ST-elevation myocardial infarction (STEMI) - represents the most dramatic and life-threatening manifestation of coronary artery disease and is diagnosed on the basis of clinical presentation, electrocardiographic changes, and cardiac biomarkers [2]. Large multinational cohorts have shown that cardiovascular risk and event rates vary substantially across income settings, with low- and middle-income countries bearing an increasing share of the global burden [3].

India illustrates this trend starkly: coronary artery disease accounts for roughly one-fourth of all deaths in the country [4], and Indians tend to develop ACS 5-10 years earlier than Western populations, often with more extensive disease and worse outcomes [5]. This early and aggressive presentation has been attributed to a distinctive constellation of risk factors - central obesity, insulin resistance, dyslipidemia, and a pro-inflammatory state - collectively termed the “Asian Indian phenotype” [6]. BMI, the most widely used anthropometric screening tool, correlates poorly with these underlying abnormalities: it cannot distinguish fat mass from lean mass and does not capture the distribution of body fat, so individuals with a normal BMI may nonetheless carry substantial visceral adiposity and cardiometabolic risk [7].

Recognition of this heterogeneity has given rise to the concept of obesity phenotypes, which integrate BMI with metabolic status to classify individuals as metabolically healthy normal weight (MHNW), metabolically unhealthy normal weight (MUNW), metabolically healthy overweight/obese (MHO), or metabolically unhealthy overweight/obese (MUO) [8]. Metabolically unhealthy phenotypes - regardless of BMI category - are characterized by insulin resistance, dyslipidemia, and hypertension and carry disproportionately higher cardiovascular risk; the MUNW phenotype in particular is common in Asian populations and frequently escapes detection because BMI appears normal.

In parallel, several novel anthropometric indices have been developed to better capture central adiposity and body shape than BMI or waist circumference alone. Waist-to-height ratio (WHtR), with a proposed universal cut-off of 0.5, is a simple index of central obesity that has outperformed BMI in several cardiometabolic outcome studies [9]. The body roundness index (BRI) estimates body fat and visceral adiposity from waist circumference and height and correlates more closely with cardiometabolic risk factors than BMI [10]. A body shape index (ABSI) was designed to quantify abdominal adiposity independent of body size and predicts all-cause and cardiovascular mortality even after adjustment for BMI [11]. The weight-adjusted waist index and abdominal volume index similarly incorporate waist circumference to estimate central adiposity independent of overall body weight.

Despite growing global evidence linking obesity phenotypes and novel anthropometric indices to cardiovascular outcomes, data specific to Indian patients presenting with ACS remain scarce. Most Indian studies have relied on BMI-based classification of obesity or the metabolic syndrome without systematically examining phenotype-based risk stratification or comparing the discriminatory performance of novel anthropometric indices against conventional measures. Given the high prevalence of central obesity, insulin resistance, and premature coronary events in India, clarifying these associations could facilitate earlier identification of high-risk individuals who are missed by BMI-based screening alone. We therefore undertook this hospital-based case-control study to evaluate the association between cardiometabolic risk factors, obesity phenotypes, and novel anthropometric indices in patients with ACS, and to quantify the strength of these associations relative to conventional risk assessment.

## Materials and methods

Study design and setting

This hospital-based case-control study was conducted in the Department of General Medicine, NKP Salve Institute of Medical Sciences and Research Center and Lata Mangeshkar Hospital, Nagpur, Maharashtra, India, from May 1, 2024, to December 31, 2025 including the general medicine wards, intensive coronary care unit, and medical intensive care unit of a tertiary care teaching hospital. A case-control design was chosen to allow comparison of exposure to cardiometabolic and anthropometric risk factors between patients with ACS and matched controls without ACS. The study was approved by the Institutional Ethics Committee and conducted in accordance with the principles of the Declaration of Helsinki; written informed consent was obtained from all participants prior to enrolment.

Sample size calculation

The sample size was calculated a priori at a 95% confidence level and 80% power, based on effect sizes reported in previously published literature on obesity phenotypes and cardiovascular risk [12]. The exact expected proportions, effect-size estimate, and formula used in this calculation were not fully retained in the study's protocol documentation and cannot be reported with certainty; this is acknowledged as a limitation. Considering feasibility and the time constraints of a single-center study, a total of 100 participants - 50 cases and 50 controls - were recruited.

Participants

Cases were adults aged 18 years or older admitted with a confirmed diagnosis of ACS (unstable angina, NSTEMI, or STEMI), established on clinical evaluation, electrocardiographic findings, and cardiac biomarkers. Controls were apparently healthy adults aged 18 years or older, recruited from hospital staff, attendants, or visitors with no history or clinical evidence of ACS. Controls were group- (frequency-) matched to the case series rather than individually paired to a specific case: recruitment targeted a control group with an overall age distribution, sex distribution, and mean body weight comparable to that of the cases, without a predefined numerical tolerance band and without 1:1 pairing. Because matching was performed at the group level, all comparisons were analyzed as independent samples (t-test, chi-square, and unconditional odds ratios) rather than with matched-pair statistical methods; this is discussed further as a limitation, particularly with respect to its potential to attenuate observed differences in BMI, which was itself a matching variable, relative to indices of central adiposity that were not directly matched upon. Patients with end-stage renal disease, chronic liver disease, hypothyroidism, advanced chronic respiratory illness, malignancy, any terminal illness, or critical illness precluding anthropometric assessment were excluded from both groups; the same exclusion criteria were applied to cases and controls. Participants were enrolled by consecutive convenience sampling until the target sample size was reached, after providing written informed consent. Participants who withdrew consent, or in whom complete clinical, anthropometric, or biochemical data could not be obtained, were withdrawn from analysis.

Data collection

Data were collected using a predesigned case record form. Sociodemographic details (age, sex, education, occupation, and income) were recorded, and socioeconomic status was graded using the Modified Kuppuswamy scale [13]. History of hypertension, diabetes mellitus, dyslipidemia, ischemic heart disease, and family history of cardiovascular disease was documented, along with smoking status, alcohol use, dietary pattern, and physical activity level. A general physical examination and systemic examination, with particular attention to the cardiovascular and respiratory systems, was performed for all participants, including screening for clinical signs of insulin resistance (e.g., acanthosis nigricans) and heart failure.

Procedure

Anthropometric measurements were obtained by a single investigator using standardized techniques to minimize inter-observer variability. Height was measured with a stadiometer and weight with a calibrated digital scale; BMI was calculated as weight (kg) divided by height squared (m²) and categorized as normal (18.5-24.9 kg/m²), overweight (25.0-29.9 kg/m²), or obese (≥30 kg/m²) [14]. Waist circumference was measured midway between the lowest rib and the iliac crest at the end of normal expiration, and hip circumference at the level of the greater trochanters; waist-to-hip ratio was calculated from these values. Neck circumference was measured just below the laryngeal prominence with the participant's head in the Frankfort horizontal plane. Blood pressure was recorded in the sitting position after rest, as the mean of two readings taken five minutes apart.

For cases, all anthropometric measurements and venous blood sampling were performed at the time of hospital admission for the index ACS event, typically within the first 24-48 hours of presentation; consequently, biochemical parameters in cases reflect the peri-admission period rather than a documented pre-morbid baseline. Venous blood samples were drawn after an overnight fast of 8-10 hours for estimation of fasting blood glucose, lipid profile, serum uric acid, and C-reactive protein; postprandial glucose was measured two hours after a standard meal. Hypertension was defined as systolic blood pressure ≥140 mmHg and/or diastolic blood pressure ≥90 mmHg or current antihypertensive use [15]; diabetes mellitus as fasting glucose ≥126 mg/dL and/or postprandial glucose ≥200 mg/dL or current antidiabetic medication use [16], based on admission values supplemented by documented pre-existing diagnosis and current antidiabetic medication where available to reduce misclassification of stress hyperglycemia as diabetes; and dyslipidemia according to Adult Treatment Panel III criteria [17]. A 12-lead electrocardiogram was recorded for all cases, and echocardiography was performed where clinically indicated.

Novel anthropometric indices were computed from these measurements using standard formulae, with waist circumference (WC) and height (Ht) in centimeters and body weight (Wt) in kilograms unless otherwise specified: WHtR (dimensionless) as WC(cm) divided by Ht(cm) [9]; ABSI (units: m¹ᵢ⁶·kg⁻²ᵢ³, reported here in the same relative units as the source formula) as WC(m) divided by the product of BMI²ᐟ³(kg/m²)²ᐟ³ and height¹ᐟ²(m)¹ᐟ² [11]; BRI (dimensionless) derived from WC and Ht using an ellipse-based eccentricity model [10]; AVI (in cm², derived from WC and hip circumference in cm) from waist and hip circumferences [18]; and the weight-adjusted waist index (WWI, in cm/√kg) as WC(cm) divided by the square root of body weight(kg) [19]. Based on BMI category and metabolic status, participants were classified into the four obesity phenotypes described above: "metabolically unhealthy" was defined as the presence of one or more of hypertension, diabetes mellitus, or dyslipidemia, and "metabolically healthy" as the absence of all three, combined with BMI category (normal weight vs overweight/obese) to yield MHNW, MUNW, MHO, and MUO [8,20].

Outcome measures

The primary outcome was the association between cardiometabolic risk factors - including hypertension, diabetes mellitus, dyslipidemia, and smoking - and obesity-related parameters, including BMI, waist circumference, neck circumference, waist-to-hip ratio, obesity phenotype, and novel anthropometric indices, with the presence of ACS. No secondary outcomes were prespecified for this study.

Statistical analysis

Data were compiled and analyzed using IBM SPSS Statistics (IBM Corp., Armonk, NY). Categorical variables were expressed as frequencies and percentages and compared using the chi-square test, or Fisher's exact test where expected cell counts were below five; unadjusted odds ratios (ORs) with 95% confidence intervals (CIs) for categorical risk factors and obesity phenotypes were calculated directly from the corresponding 2×2 contingency tables.

For continuous variables, group means were first compared using the independent-samples t-test. Each continuous variable was then standardized to a mean of 0 and a standard deviation (SD) of 1 (i.e., z-scored using its pooled SD across cases and controls) and entered individually into a univariable binary logistic regression with ACS status as the outcome, so that the reported estimate corresponds directly to a one-SD increase in the original variable. This standardization was applied uniformly to all continuous exposures (BMI, waist circumference, waist-to-hip ratio, neck circumference, ABSI, BRI, WHtR, WWI, and AVI) because several of these measures (in particular waist-to-hip ratio, ABSI, and WHtR) vary by only a few hundredths across the study population, making a one-native-unit increment physiologically implausible; standardizing before fitting the regression, rather than rescaling an already-fitted native-unit estimate, ensures that the reported coefficient is a direct output of the regression on the actual variable used, not a post hoc transformation of it. For each standardized variable, we report the exponentiated regression coefficient, Exp(B), with its 95% CI, the Wald statistic, and the associated p-value, exactly as produced by the univariable logistic regression. Multivariable regression to adjust for potential confounders (e.g., age, smoking, comorbidities) was not performed, and these unadjusted estimates should be interpreted as measures of association rather than as evidence of an independent or causal effect. Because the anthropometric indices differ in what they geometrically represent, their per-SD estimates are not used to rank the indices by discriminative strength, and no formal test of discriminative performance (e.g., receiver operating characteristic curve/area-under-the-curve analysis) was undertaken. A two-tailed p-value <0.05 was considered statistically significant. Test statistics reported alongside p-values in the Results tables (t for continuous group comparisons, χ² for categorical comparisons, and the Wald statistic for logistic regression-based and odds ratio-based associations) are presented in a dedicated column separate from the corresponding p-value.

## Results

A total of 100 participants were enrolled, comprising 50 cases with ACS and 50 age-, sex-, and weight-matched controls. Test statistics (t for continuous variables, χ² for categorical variables, and Wald χ² for odds ratio-based associations) are reported in a separate column alongside the corresponding p-value in each table.

The mean age of cases (56.4 ± 9.8 years) was comparable to that of controls (55.9 ± 10.2 years), with no significant difference (Table 1). Most participants in both groups were 41-60 years old, and males predominated in both cases and controls (72%). The lack of significant difference in age and sex distribution confirms appropriate matching between groups.

**Table 1:** Distribution of study participants according to age and sex. SD: Standard deviation. Test statistics were computed from reported summary data using the independent-samples t-test (continuous variables) or the chi-square test (categorical variables).

Variable	Cases (n=50)	Controls (n=50)	Test statistic	p-Value
Age (years), mean ± SD	56.4 ± 9.8	55.9 ± 10.2	t = 0.25	0.803
Age group 18-40 years	6 (12%)	7 (14%)	χ² = 0.10	0.952
Age group 41-60 years	24 (48%)	23 (46%)	(df = 2)	
Age group >60 years	20 (40%)	20 (40%)		
Male sex	36 (72%)	36 (72%)	χ² = 0.00	1
Female sex	14 (28%)	14 (28%)	(df = 1)	

Education, occupation, and socioeconomic status were comparable between cases and controls, with no significant differences (Table 2), indicating that sociodemographic disparity is unlikely to confound the associations examined.

**Table 2:** Distribution of study participants according to sociodemographic characteristics. SES: Socioeconomic status (Modified Kuppuswamy scale). Rows are grouped by education, occupation, and SES; the test statistic and p-value apply to the overall comparison of each grouped variable.

Sociodemographic variable	Cases (n=50)	Controls (n=50)	Test statistic	p-Value
Illiterate	18 (36%)	14 (28%)	χ² = 1.59	0.663
Primary school	16 (32%)	14 (28%)	(df = 3)	
Secondary school	10 (20%)	14 (28%)		
Graduate and above	6 (12%)	8 (16%)		
Unemployed / homemaker	16 (32%)	14 (28%)	χ² = 0.70	0.874
Unskilled worker	18 (36%)	16 (32%)	(df = 3)	
Semi-skilled worker	8 (16%)	10 (20%)		
Skilled / professional	8 (16%)	10 (20%)		
Upper SES	2 (4%)	4 (8%)	χ² = 1.81	0.771
Upper-middle SES	8 (16%)	10 (20%)	(df = 4)	
Lower-middle SES	14 (28%)	16 (32%)		
Upper-lower SES	18 (36%)	14 (28%)		
Lower SES	8 (16%)	6 (12%)		

Current smoking was significantly more common among cases than controls (44% vs 24%; Table 3), while sedentary behavior, though numerically higher in cases (56% vs 36%), narrowly missed the conventional significance threshold on chi-square testing across the three-category physical activity variable. Alcohol use and dietary pattern did not differ significantly between groups.

**Table 3:** Comparison of lifestyle risk factors between cases and controls. *Statistically significant (p<0.05).

Lifestyle risk factor	Cases (n=50)	Controls (n=50)	Test statistic	p-Value
Current smoker	22 (44%)	12 (24%)	χ² = 6.16	0.046*
Ex-smoker	10 (20%)	8 (16%)	(df = 2)	
Never smoker	18 (36%)	30 (60%)		
Current alcohol user	20 (40%)	12 (24%)	χ² = 2.94	0.086
Non-alcoholic	30 (60%)	38 (76%)	(df = 1)	
Sedentary	28 (56%)	18 (36%)	χ² = 4.12	0.127
Moderately active	16 (32%)	22 (44%)	(df = 2)	
Physically active	6 (12%)	10 (20%)		
Mixed diet	38 (76%)	32 (64%)	χ² = 1.71	0.19
Vegetarian diet	12 (24%)	18 (36%)	(df = 1)	

Hypertension, diabetes mellitus, and dyslipidemia were each significantly more prevalent among cases than controls (Table 4). A past history of ischemic heart disease was present only among cases and showed a highly significant association (p<0.001, Fisher's exact test), while family history of cardiovascular disease showed a similar but non-significant trend.

**Table 4:** Comparison of clinical characteristics and comorbidities between cases and controls. CVD: Cardiovascular disease; IHD: Ischemic heart disease. * Statistically significant (p<0.05).

Clinical risk factor	Cases (n=50)	Controls (n=50)	Test statistic	p-Value
Hypertension	30 (60%)	18 (36%)	χ² = 5.77 (df = 1)	0.016*
Diabetes mellitus	26 (52%)	14 (28%)	χ² = 6.00 (df = 1)	0.014*
Dyslipidemia	28 (56%)	16 (32%)	χ² = 5.84 (df = 1)	0.016*
Family history of CVD	18 (36%)	10 (20%)	χ² = 3.17 (df = 1)	0.075
Past history of IHD	12 (24%)	0 (0%)	Fisher's exact	<0.001*

Both systolic and diastolic blood pressure were significantly higher among cases than controls (Table 5), consistent with the higher proportion of hypertensive individuals among cases.

**Table 5:** Comparison of blood pressure parameters between cases and controls. BP: Blood pressure; SD: Standard deviation. * Statistically significant (p<0.05).

Blood pressure parameter	Cases (n=50)	Controls (n=50)	Test statistic	p-Value
Systolic BP (mmHg), mean ± SD	142.6 ± 18.4	128.2 ± 14.6	t = 4.33	<0.001*
Diastolic BP (mmHg), mean ± SD	88.4 ± 10.2	80.6 ± 8.8	t = 4.09	<0.001*
Participants with hypertension	30 (60%)	18 (36%)	χ² = 5.77	0.016*

Fasting and postprandial glucose levels were significantly higher among cases (Table 6), reflecting a greater burden of impaired glucose metabolism in this group.

**Table 6:** Comparison of glycemic parameters between cases and controls. * Statistically significant (p<0.05).

Glycemic parameter	Cases (n=50)	Controls (n=50)	Test statistic	p-Value
Fasting blood glucose (mg/dL)	136.8 ± 42.6	108.4 ± 26.2	t = 4.02	<0.001*
Postprandial glucose (mg/dL)	198.6 ± 68.4	148.2 ± 42.8	t = 4.42	<0.001*
Participants with diabetes mellitus	26 (52%)	14 (28%)	χ² = 6.00	0.014*

Total cholesterol, LDL cholesterol, and triglycerides were significantly higher, and HDL cholesterol significantly lower, among cases compared with controls (Table 7), consistent with a more atherogenic lipid profile in patients with ACS.

**Table 7:** Comparison of lipid profile parameters between cases and controls. LDL: Low-density lipoprotein; HDL: High-density lipoprotein. * Statistically significant (p<0.05).

Lipid parameter	Cases (n=50)	Controls (n=50)	Test statistic	p-Value
Total cholesterol (mg/dL)	212.4 ± 38.6	184.6 ± 32.4	t = 3.90	<0.001*
LDL cholesterol (mg/dL)	138.2 ± 34.8	112.6 ± 28.4	t = 4.03	<0.001*
HDL cholesterol (mg/dL)	38.6 ± 8.4	44.8 ± 9.2	t = 3.52	<0.001*
Triglycerides (mg/dL)	178.4 ± 62.6	142.8 ± 48.2	t = 3.19	0.002*
Participants with dyslipidemia	28 (56%)	16 (32%)	χ² = 5.84	0.016*

C-reactive protein and serum uric acid levels were significantly elevated among cases (Table 8). As blood was drawn at the time of admission for the acute event, elevated C-reactive protein in cases may reflect the acute-phase response to myocardial injury and ischemia rather than antecedent chronic inflammatory risk; this cross-sectional comparison cannot distinguish between the two, and pre-event C-reactive protein values were not available.

**Table 8:** Comparison of inflammatory and metabolic markers between cases and controls. * Statistically significant (p<0.05).

Parameter	Cases (n=50)	Controls (n=50)	Test statistic	p-Value
C-reactive protein (mg/L)	8.6 ± 4.2	3.2 ± 1.8	t = 8.36	<0.001*
Serum uric acid (mg/dL)	6.8 ± 1.6	5.4 ± 1.2	t = 4.95	<0.001*

No significant differences were observed between cases and controls with respect to height, weight, BMI, or the proportions of overweight/obese participants (Table 9), suggesting that BMI alone was not associated with ACS status in this cohort.

**Table 9:** Comparison of conventional anthropometric measurements between cases and controls. BMI: Body mass index.

Anthropometric parameter	Cases (n=50)	Controls (n=50)	Test statistic	p-Value
Height (cm)	164.8 ± 8.6	165.6 ± 8.2	t = 0.48	0.635
Weight (kg)	70.4 ± 11.2	69.8 ± 10.6	t = 0.28	0.784
Body mass index (kg/m²)	26.1 ± 3.8	25.4 ± 3.6	t = 0.95	0.347
Overweight (BMI 25-29.9)	24 (48%)	22 (44%)	χ² = 0.16	0.688
Obese (BMI ≥30)	8 (16%)	6 (12%)	χ² = 0.33	0.564

Waist circumference, waist-to-hip ratio, and neck circumference were all significantly higher among cases, whereas hip circumference did not differ significantly (Table 10), highlighting central and upper-body adiposity - rather than overall body size - as relevant to ACS risk.

**Table 10:** Comparison of body fat distribution parameters between cases and controls. * Statistically significant (p<0.05).

Parameter	Cases (n=50)	Controls (n=50)	Test statistic	p-Value
Waist circumference (cm)	96.8 ± 9.4	90.2 ± 8.6	t = 3.66	<0.001*
Hip circumference (cm)	100.6 ± 8.8	99.2 ± 8.4	t = 0.81	0.418
Waist-to-hip ratio	0.96 ± 0.06	0.91 ± 0.05	t = 4.53	<0.001*
Neck circumference (cm)	38.6 ± 3.2	36.4 ± 2.8	t = 3.66	<0.001*

All five novel anthropometric indices were significantly higher among cases than controls (Table 11). Because BMI did not differ significantly between groups (Table 9) while each of these indices did, this pattern is consistent with central adiposity, rather than overall body size, being relevant to ACS status in this cohort; as the indices are measured on different native scales, this comparison does not by itself establish a ranking of discriminative performance among them.

**Table 11:** Comparison of novel anthropometric indices between cases and controls. * Statistically significant (p<0.05).

Novel anthropometric index	Cases (n=50)	Controls (n=50)	Test statistic	p-Value
A Body Shape Index (ABSI)	0.083 ± 0.006	0.079 ± 0.005	t = 3.62	<0.001*
Body Roundness Index (BRI)	5.2 ± 1.1	4.4 ± 0.9	t = 3.98	<0.001*
Waist-to-height ratio (WHtR)	0.59 ± 0.06	0.54 ± 0.05	t = 4.53	<0.001*
Weight-adjusted waist index (WWI)	11.4 ± 1.2	10.2 ± 1.0	t = 5.43	<0.001*
Abdominal volume index (AVI)	17.8 ± 3.6	14.6 ± 3.2	t = 4.70	<0.001*

The MUO phenotype was most common among cases (40%), while MHNW and MHO were equally most common among controls (32% each). The overall distribution did not differ significantly between groups at the conventional threshold (Table 12), although the pattern was directionally consistent with a higher burden of metabolically unhealthy phenotypes among cases.

**Table 12:** Distribution of obesity phenotypes among cases and controls *Statistically significant (p<0.05).

Obesity phenotype	Cases (n=50)	Controls (n=50)	Test statistic	p-Value
Metabolically healthy normal weight (MHNW)	8 (16%)	16 (32%)	χ² = 6.24	0.101
Metabolically unhealthy normal weight (MUNW)	10 (20%)	6 (12%)	(df = 3)	
Metabolically healthy overweight/obese (MHO)	12 (24%)	16 (32%)		
Metabolically unhealthy overweight/obese (MUO)	20 (40%)	12 (24%)		

Using MHNW as the reference category, the MUO phenotype showed significantly increased odds of ACS (OR 3.33, 95% CI 1.10-10.12), whereas MUNW showed a similarly sized point estimate (OR 3.33) that did not reach statistical significance (95% CI 0.89-12.49; p=0.074), and MHO showed no significant association (Table 13). The wide confidence intervals, particularly for MUNW, reflect the small cell counts in this phenotype-stratified analysis and limit the precision of these estimates; these findings should be regarded as hypothesis-generating rather than conclusive regarding the relative risk of the metabolically unhealthy normal-weight phenotype.

**Table 13:** Association between obesity phenotypes and acute coronary syndrome. OR: Odds ratio; CI: Confidence interval; MHNW: Metabolically healthy normal weight (reference category); MUNW: Metabolically unhealthy normal weight; MHO: Metabolically healthy overweight/obese; MUO: Metabolically unhealthy overweight/obese. Wald statistic derived from the OR and its 95% CI. * Statistically significant (p<0.05).

Obesity phenotype	Cases	Controls	OR (95% CI)	Wald χ²	p-Value
MHNW (reference)	8	16	1	—	—
MUNW	10	6	3.33 (0.89–12.49)	3.19	0.074
MHO	12	16	1.50 (0.48–4.65)	0.49	0.483
MUO	20	12	3.33 (1.10–10.12)	4.52	0.033*

Hypertension, diabetes mellitus, dyslipidemia, and current smoking were each significantly associated with ACS (Table 14), while family history showed a similar direction of effect without reaching significance.

**Table 14:** Association of traditional cardiometabolic risk factors with acute coronary syndrome. OR: Odds ratio; CI: Confidence interval; CVD: Cardiovascular disease. *Statistically significant (p<0.05).

Risk factor	Cases	Controls	OR (95% CI)	Wald χ²	p-Value
Hypertension	30	18	2.67 (1.19–6.01)	5.65	0.017*
Diabetes mellitus	26	14	2.78 (1.22–6.34)	5.91	0.015*
Dyslipidemia	28	16	2.69 (1.20–6.04)	5.76	0.016*
Current smoking	22	12	2.46 (1.03–5.88)	4.1	0.043*
Family history of CVD	18	10	2.25 (0.92–5.49)	3.17	0.075

BMI showed no significant association with ACS per 1-SD increase, whereas waist circumference, waist-to-hip ratio, and neck circumference - all measures of central or upper-body adiposity - were significantly associated with increased odds of ACS per 1-SD increase (Table 15).

**Table 15:** Association of conventional anthropometric parameters with acute coronary syndrome (standardized univariable logistic regression). Values are presented as mean ± standard deviation (SD) for cases and controls. Odds ratios are expressed as Exp(B) per 1-SD increase in the respective anthropometric parameter, with corresponding 95% confidence intervals (CIs). BMI: Body mass index; SD: Standard deviation; Exp(B): Exponentiated regression coefficient (odds ratio); CI: Confidence interval; Wald: Wald test statistics. P < 0.05 was considered statistically significant; P < 0.001 is indicated by an asterisk.

Anthropometric parameter	Mean ± SD (cases)	Mean ± SD (controls)	Exp(B) per 1-SD (95% CI)	Wald χ²	p-Value
Body mass index (kg/m²)	26.1 ± 3.8	25.4 ± 3.6	1.208 (0.816–1.790)	0.89	0.345
Waist circumference (cm)	96.8 ± 9.4	90.2 ± 8.6	2.081 (1.388–3.119)	12.57	<0.001*
Waist-to-hip ratio	0.96 ± 0.06	0.91 ± 0.05	2.473 (1.638–3.732)	18.59	<0.001*
Neck circumference (cm)	38.6 ± 3.2	36.4 ± 2.8	2.079 (1.387–3.116)	12.55	<0.001*

Each of the five novel anthropometric indices showed a statistically significant unadjusted association with ACS per 1-SD increase (Table 16). Because these estimates are unadjusted for other covariates, they cannot be used to conclude that any one index is independently or causally related to ACS; standardized, covariate-adjusted analyses fitted directly on individual-level data in a larger dataset would be required to support such conclusions. Standardizing all continuous exposures to a common per-1-SD scale before fitting each regression, rather than fitting on each index's own native unit and rescaling afterward, ensures that the reported estimates are internally consistent and directly interpretable, including for ABSI, waist-to-hip ratio, and waist-to-height ratio, whose native ranges are too narrow for a whole-unit increment to be meaningful; nonetheless, because the indices differ in what they geometrically represent, this study still does not rank them by strength of association.

**Table 16:** Association of novel anthropometric indices with acute coronary syndrome (standardized univariable logistic regression). Values are presented as mean ± standard deviation (SD) for cases and controls. Odds ratios are expressed as Exp(B) per 1-SD increase in the respective anthropometric index, with corresponding 95% confidence intervals (CIs). ABSI: A body shape index; BRI: Body roundness index; WHtR: Waist-to-height ratio; WWI: Weight-adjusted waist index; AVI: Abdominal volume index; SD: Standard deviation; Exp(B): Exponentiated regression coefficient (odds ratio); CI: Confidence interval; Wald: Wald test statistic. P < 0.05 was considered statistically significant; P < 0.001 is indicated by an asterisk.

Novel anthropometric index	Mean ± SD (cases)	Mean ± SD (controls)	Exp(B) per 1-SD (95% CI)	Wald χ²	p-Value
ABSI	0.083 ± 0.006	0.079 ± 0.005	2.063 (1.377–3.092)	12.31	<0.001*
BRI	5.2 ± 1.1	4.4 ± 0.9	2.217 (1.475–3.331)	14.68	<0.001*
WHtR	0.59 ± 0.06	0.54 ± 0.05	2.473 (1.638–3.732)	18.59	<0.001*
WWI	11.4 ± 1.2	10.2 ± 1.0	2.964 (1.947–4.510)	25.71	<0.001*
AVI	17.8 ± 3.6	14.6 ± 3.2	2.559 (1.693–3.867)	19.88	<0.001*

## Discussion

In this hospital-based case-control study, cases and controls had comparable age, sex, and sociodemographic distributions, which reduces the likelihood that these factors confound the associations of interest, although residual confounding from unmeasured or imperfectly matched variables cannot be excluded given the group-level (rather than individually paired) matching approach used. Traditional cardiometabolic risk factors - hypertension, diabetes mellitus, dyslipidemia, and current smoking - were each significantly more prevalent among patients with ACS, consistent with findings from the landmark INTERHEART study, which identified these factors, together with abdominal obesity, as accounting for the majority of the population-attributable risk of myocardial infarction across diverse populations [21]. The magnitude of association observed for hypertension (OR 2.67), diabetes mellitus (OR 2.78), dyslipidemia (OR 2.69), and smoking (OR 2.46) in the present cohort closely parallels the higher burden of these risk factors reported at younger ages among Indians with premature coronary artery disease [5]. Similarly, Indian epidemiological data have documented a high prevalence of hypertension, diabetes, and dyslipidemia in the population at large, broadly consistent with the elevated prevalence of these factors among cases in the present study [4]. For context, a comparable pattern of elevated cardiometabolic comorbidity has been reported in a different cardiac population: among patients presenting with a dilated cardiomyopathy phenotype undergoing coronary angiography, those with obstructive coronary artery disease had a significantly higher prevalence of diabetes mellitus (42.1% vs 15.9%) and dyslipidemia than those without obstructive disease [22]; although that study examined a different clinical population and cannot be directly compared with the present ACS cohort, it lends broader support to the recurring association between these cardiometabolic comorbidities and obstructive coronary disease across cardiac presentations. Although family history of cardiovascular disease showed a similar direction of effect, it did not reach statistical significance, which may reflect the modest sample size rather than a true absence of association, given that a positive family history has consistently been linked to increased cardiovascular risk in larger epidemiological cohorts.

Inflammatory and metabolic markers were also significantly elevated among cases, but their interpretation in this study requires caution because of when they were measured. C-reactive protein, a marker of vascular inflammation implicated in plaque instability and atherogenesis, was markedly higher among cases, in keeping with prospective evidence linking elevated C-reactive protein to increased cardiovascular risk in general populations [23]. However, because blood was drawn at the time of admission for the acute event rather than before it, elevated C-reactive protein in cases most plausibly reflects, at least in part, the acute-phase response to myocardial injury and ischemic stress rather than antecedent chronic inflammatory risk; this cross-sectional, post-event measurement cannot distinguish between the two, and pre-event C-reactive protein values were not available for comparison. A similar caveat applies to glycemic classification: admission glucose values in the acute setting can be elevated by stress hyperglycemia independent of underlying diabetes status, and while diabetes classification in this study incorporated documented pre-existing diagnosis and antidiabetic medication use where available, some misclassification of stress hyperglycemia as diabetes mellitus cannot be excluded, which may in turn have affected obesity phenotype assignment for a subset of cases. Serum uric acid was likewise elevated among cases; whether this reflects a pre-existing metabolic abnormality or an acute correlate of the ischemic event itself cannot be determined from this design. These findings should therefore be interpreted as describing the metabolic and inflammatory profile present at the time of ACS presentation rather than as establishing antecedent causal risk.

A key finding of this study is that BMI did not differ significantly between cases and controls, despite comparable proportions of overweight and obesity in both groups, and showed no significant association with ACS per 1-SD increase on standardized univariable logistic regression. This is consistent with mounting evidence that BMI, as a global measure of adiposity, does not adequately capture body fat distribution or metabolic risk, particularly in South Asians, who tend to accumulate visceral fat and develop cardiometabolic abnormalities at lower BMI thresholds than White populations [24]; the World Health Organization has, accordingly, proposed lower BMI cut-offs for overweight and obesity in Asian populations to better reflect this elevated risk at a given BMI [25]. It should also be noted that controls were recruited to approximate the weight distribution of cases; because weight, but not waist circumference or the other indices evaluated, was a target of this group-level matching, the null BMI finding should be interpreted with caution, as matching on a correlate of BMI may have attenuated between-group BMI differences relative to indices of central adiposity that were not directly matched upon. In contrast, waist circumference, waist-to-hip ratio, and neck circumference - measures that capture central and upper-body fat distribution - were all significantly associated with ACS per 1-SD increase in this cohort. This pattern mirrors findings from Indian urban cohorts, in which waist circumference and waist-to-hip ratio showed stronger correlation with cardiovascular risk factors than BMI [26], and from the Framingham Heart Study, which identified neck circumference as a correlate of cardiometabolic risk that added information beyond BMI and waist circumference [27]. The INTERHEART study similarly reported that abdominal obesity, assessed by waist-to-hip ratio, carried a substantially higher population-attributable risk for myocardial infarction than BMI across ethnic groups, including South Asians [28].

Each of the five novel anthropometric indices evaluated - ABSI, BRI, WHtR, WWI, and AVI - was significantly higher among cases and showed a statistically significant unadjusted association with ACS per 1-SD increase on standardized univariable logistic regression. These findings are consistent with the rationale underlying the development of each index: WHtR, incorporating waist circumference relative to height, has been proposed as a simple screening tool for cardiometabolic risk [9]; BRI, modeled on waist circumference and height, correlates with visceral adiposity and metabolic risk factors [10]; and ABSI, designed to capture abdominal adiposity independent of body size, has been shown in other cohorts to predict cardiovascular and all-cause mortality after adjustment for BMI and waist circumference [11]. Because these estimates are unadjusted for other covariates, their relative magnitudes cannot be used to rank the indices or to conclude that any one index is superior to the others, even though all are now expressed on a common per-1-SD scale; each index represents a conceptually distinct geometric construct, and a meaningful ranking would additionally require formal discrimination analyses (e.g., receiver operating characteristic curves) fitted on individual-level, multivariable-adjusted data, which was beyond the scope of this study. The consistent elevation of these indices among cases nonetheless suggests a possible complementary role alongside BMI in cardiovascular risk assessment, particularly in populations such as Asian Indians who develop visceral obesity at lower BMI thresholds, a hypothesis that warrants prospective evaluation.

The analysis of obesity phenotypes provides further insight into this relationship. Using the metabolically healthy normal-weight phenotype as reference, the MUO phenotype showed significantly increased odds of ACS (OR 3.33, 95% CI 1.10-10.12), consistent with meta-analytic evidence that the combination of excess adiposity and metabolic abnormality is associated with a higher cardiovascular risk than either factor alone [29]. The MUNW phenotype - individuals with normal BMI but underlying metabolic dysfunction - showed a point estimate of similar magnitude (OR 3.33) but, with a wider confidence interval (95% CI 0.89-12.49), did not reach conventional statistical significance (p=0.074) in this sample; given the small cell counts involved, this finding is better regarded as a trend consistent with, rather than confirmatory of, prior recognition that metabolically unhealthy normal-weight individuals, sometimes described as having a “thin-fat” phenotype, may carry cardiovascular risk approaching that of individuals with overt obesity and metabolic disease [8]; a larger sample would be needed to determine whether this association reaches significance. In contrast, the MHO phenotype was not significantly associated with ACS in this cohort, in keeping with evidence that metabolically healthy obesity, at least in the short term, is associated with a lower cardiovascular risk than metabolically unhealthy phenotypes; however, this state has also been described as unstable over time in a substantial proportion of individuals, with transition toward metabolic unhealthiness and a corresponding rise in cardiovascular risk [30]. Collectively, these phenotype-based findings are directionally consistent with the view that metabolic health, in addition to body size, is relevant to cardiovascular risk assessment, although only the MUO comparison reached statistical significance in this sample; because phenotype assignment relies in part on admission-based diagnoses that may be subject to the acute-phase and stress-hyperglycemia considerations noted above, and because these odds ratios are likewise unadjusted, this observation should be regarded as hypothesis-generating rather than conclusive.

This study has several limitations, in addition to those noted above regarding measurement timing and matching. The single-center design and modest sample size (50 cases and 50 controls) limit statistical power, particularly for phenotype subgroup comparisons, and likely explain the wide confidence intervals and non-significant associations observed for the MUNW phenotype, MHO phenotype, family history, and alcohol use. All effect estimates presented are unadjusted; multivariable regression to account for potential confounders (including age, smoking, and comorbidities) was not performed, and these unadjusted estimates should be interpreted as measures of association rather than evidence of an independent or causal effect. Because the anthropometric indices differ in what they geometrically represent, this study does not draw conclusions about their relative discriminative superiority even though all are now expressed on a common per-1-SD scale; formal comparison would require discrimination analyses (e.g., receiver operating characteristic curves) fitted on individual-level, multivariable-adjusted data, which were not performed. The case-control design precludes assessment of temporal or causal relationships between obesity phenotypes, novel anthropometric indices, and ACS, and post-event measurement of inflammatory and glycemic markers limits their interpretation as antecedent risk factors, as discussed above. Direct imaging-based measures of visceral adiposity and biomarkers of insulin resistance were not assessed, which would have strengthened mechanistic interpretation. Convenience sampling of controls from hospital attendants and staff, together with group-level rather than individually paired matching, may not fully represent the general population and may have introduced selection bias or attenuated some between-group differences, particularly for BMI. Larger, multicentric, prospective studies with individual-level data available for direct multivariable modeling, imaging-based adiposity measures, and pre-event biomarker sampling are warranted to validate these findings and to establish the clinical utility of novel anthropometric indices in routine cardiovascular risk prediction models.

## Conclusions

In this hospital-based case-control study, traditional cardiometabolic risk factors and central adiposity measures - but not BMI - were significantly associated with ACS in unadjusted analyses. Obesity phenotyping suggested that the metabolically unhealthy overweight/obese phenotype was associated with significantly higher odds of ACS than the metabolically healthy normal-weight phenotype (OR 3.33), while the metabolically unhealthy normal-weight phenotype showed a similarly sized point estimate that did not reach statistical significance in this sample. Each of the five novel anthropometric indices evaluated (ABSI, BRI, WHtR, WWI, and AVI) was significantly associated with ACS status, in contrast to BMI; because these are unadjusted associations measured on different scales, this study does not establish a ranking among them or demonstrate independent or superior discriminative performance. Elevated C-reactive protein and glucose among cases should be interpreted in light of their measurement at the time of acute presentation, which limits inference about antecedent risk. Taken together, these findings support further investigation of obesity phenotyping and novel anthropometric indices - alongside conventional cardiometabolic risk factors - as potential adjuncts to cardiovascular risk assessment in South Asian populations, but larger, prospective, multivariable-adjusted studies with pre-event biomarker sampling are needed before their incremental clinical value and relative performance can be established.
